# *In vitro* Characteristics of Heterogeneous Equine Hoof Progenitor Cell Isolates

**DOI:** 10.3389/fbioe.2019.00155

**Published:** 2019-07-11

**Authors:** Qingqiu Yang, Vanessa Marigo Rocha Pinto, Wei Duan, Erica E. Paxton, Jenna H. Dessauer, William Ryan, Mandi J. Lopez

**Affiliations:** Laboratory for Equine and Comparative Orthopedic Research, Department of Veterinary Clinical Sciences, School of Veterinary Medicine, Louisiana State University, Baton Rouge, LA, United States

**Keywords:** mesoderm, ectoderm, ultrastructure, scaffold, horse, stem, keratin

## Abstract

Damage to an ectodermal-mesodermal interface like that in the equine hoof and human finger nail bed can permanently alter tissue structure and associated function. The purpose of this study was to establish and validate *in vitro* culture of primary progenitor cell isolates from the ectodermal-mesodermal tissue junction in equine hooves, the stratum internum, with and without chronic inflammation known to contribute to lifelong tissue defects. The following were evaluated in hoof stratum internum cell isolates up to 5 cell passages (P): expansion capacity by cell doublings and doubling time; plasticity with multi-lineage differentiation and colony-forming unit (CFU) frequency percentage; immunophenotype with immunocytochemistry and flow cytometry; gene expression with RT-PCR; and ultrastructure with transmission electron microscopy. The presence of keratin (K)14, 15 and K19 as well as cluster of differentiation (CD)44 and CD29 was determined *in situ* with immunohistochemistry. To confirm *in vivo* extracellular matrix (ECM) formation, cell-scaffold (polyethylene glycol/poly-L-lactic acid and tricalcium phosphate/hydroxyapatite) constructs were evaluated with scanning electron microscopy 9 weeks after implantation in athymic mice. Cultured cells had characteristic progenitor cell morphology, expansion, CFU frequency percentage and adipocytic, osteoblastic, and neurocytic differentiation capacity. CD44, CD29, K14, K15 and K19 proteins were present in native hoof stratum internum. Cultured cells also expressed K15, K19 and desmogleins 1 and 3. Gene expression of CD105, CD44, K14, K15, sex determining region Y-box 2 (SOX2) and octamer-binding transcription factor 4 (OCT4) was confirmed *in vitro*. Cultured cells had large, eccentric nuclei, elongated mitochondria, and intracellular vacuoles. Scaffold implants with cells contained fibrous ECM 9 weeks after implantation compared to little or none on acellular scaffolds. *In vitro* expansion and plasticity and *in vivo* ECM deposition of heterogeneous, immature cell isolates from the ectodermal-mesodermal tissue interface of normal and chronically inflamed hooves are typical of primary cell isolates from other adult tissues, and they appear to have both mesodermal and ectodermal qualities *in vitro*. These results establish a unique cell culture model to target preventative and restorative therapies for ectodermal-mesodermal tissue junctions.

## Introduction

The equine hoof is a complex anatomical structure that changed in size and shape as horses evolved from a multiple to a single toe ungulate (Pollitt, 1992; O'grady, 2002; Van Eps and Pollitt, 2009). Within a keratinized hoof wall, the last phalanx of the third digit is suspended by the stratum internum (lamellatum) (Pollitt, 1998), an attachment that can be permanently and fatally disrupted by inflammation within the hoof wall known as laminitis (Johnson et al., 1998; Pollitt and Daradka, 1998; Morgan et al., 1999; Longland and Byrd, 2006; Van Eps and Pollitt, 2009). Following damage, complex hoof architecture is replaced with abundant, poorly organized tissue (Hunt and Wharton, 2010; Barreto-Vianna et al., 2013), and current treatments to restore normal tissue after disruption are largely unsuccessful (Atala et al., 2010).

Tissue formation in the stratum medium, the cornified tissue layer directly above the stratum internum, results from basal cell proliferation in the coronary band (Daradka and Pollitt, 2004) located at the proximal aspect of the structure. The cells undergo maturation into partially, keratinocyte, or fully, corneocyte, keratinized cells as they move downward from the basal layer. Cells on the surface of the redundant, frond-like lamellae that constitute the ectodermal-mesodermal tissues in the stratum internum are connected to each other by tight cell junctions (French and Pollitt, 2004; Visser and Pollitt, 2011). As the ectodermal component of the tissue connected to the outer hoof wall grows downward relative to the mesodermal component fixed to the boney third phalanx via protein fibrils, cells are thought to detach and reattach via controlled enzymatic activity, largely within the basement membrane between them (Pollitt, 1994; Daradka and Pollitt, 2004). While cell growth and maturation is well-described for the cornified hoof tissue, the role of progenitor cells within the stratum internum to production and maintenance of the deeper hoof tissues, especially those at the ectodermal-mesodermal interface, are not well-elucidated (Anderson and Mcilwraith, 2004; Visser and Pollitt, 2010).

Isolation and characterization of progenitor cells from the ectodermal-mesodermal tissue nich of normal and inflamed, laminitic, hooves is necessary to advance understanding of cell behavior and reparative capacity under normal and inflamed conditions (Lian and Stein, 1992; Kisiday et al., 2002). Proliferating cells were identified in normal hoof lamellae with 5-bromo-2'-deoxyuridine (BRdU) (Daradka and Pollitt, 2004). Additionally, epithelial progenitor cells in the lamellae were localized by K14 and transcription factor p63 expression (Senoo et al., 2007; Bragulla and Homberger, 2009; Broeckx et al., 2014; Linardi et al., 2015). This information confirms that progenitor cells exist within the normal stratum internum. Altered progenitor cell behavior from disease and chronic inflammation disrupts tissue formation and healing in ectodermal-dermal tissue interfaces like the periodontal membrane and finger nail bed (Page and Schroeder, 1976; Ding et al., 2010; Bharathi and Bajantri, 2011). A mechanism to confidently isolate and culture hoof progenitor cells from the ectodermal-mesodermal tissue niche of normal and chronically inflamed hooves will provide a useful tool to evaluate mechanisms of cell damage and target therapies to prevent cell loss and restore normal growth following injury, local disease or systemic pathology. The purpose of this study was to establish and validate *in vitro* culture of progenitor cells from the stratum internum of equine hooves with and without chronic inflammation.

## Materials and Methods

### Study Design

Forelimbs from 22 horses belonging to the University research herd, 14 unaffected (U), and 8 with laminitis (L), were disarticulated at the metacarpophalangeal joint following humane euthanasia for reasons unrelated to this study. Cells were isolated from the stratum internum and progenitor cells selected by plastic affinity. Outcome measures included cell expansion rate for cell passages (P) 1-3 (*n* = 5 U; *n* = 6 L), P1 trilineage differentiation (*n* = 3 U; *n* = 3 L), P0, 2 and 5 colony forming unit frequency (CFU, *n* = 4 U; *n* = 3 L) and cell surface marker expression (*n* = 8 U; *n* = 6 L), hoof tissue immunohistochemistry (IHC) (*n* = 2 U; *n* = 1 L), immunocytochemistry (ICC) of P1 and 3 (*n* = 2 U; *n* = 2 L), P0, 2 and 5 gene expression of CD44, CD105, K14, K15, octamer-binding transcription factor 4 (OCT4), and sex determining region Y-box 2 (SOX2) (*n* = 4 U; *n* = 5 L) and transmission electron microscopy (TEM) of P1 cell ultrastructure (*n* = 2 U; *n* = 2 L). Scanning electron microscopy (SEM, *n* = 1 U) was used to assess extracellular matrix (ECM) deposition on polyethylene glycol/poly-L-lactic acid (GA) and tricalcium phosphate/hydroxyapatite (HT) scaffolds loaded with P3 cells 9 weeks after subcutaneous implantation in athymic mice (Table 1).

**Table 1:** Study samples and assays.

**Age (year)**	**Condition**	**Gender**	**Breed**	**Outcomes**						
				**CFU**	**CD/DT**	**ICC**	**IHC**	**PCR**	**FC**	**TEM**
3	Unaffected	Gelding	Thoroughbred	X	X					
8	Unaffected	Mare	Thoroughbred	X	X					
2	Unaffected	Mare	Thoroughbred	X	X					
4	Unaffected	Gelding	Thoroughbred	X	X					
13	Unaffected	Mare	Quarter Horse		X	X				
3	Unaffected	Gelding	Thoroughbred					X	X	
14	Unaffected	Mare	Quarter Horse						X	
9	Unaffected	Gelding	Tennessee Walker					X	X	
7	Unaffected	Mare	Arabian				X	X	X	
7	Unaffected	Gelding	Paint Horse					X	X	
22	Unaffected	Gelding	Palomino						X	
17	Unaffected	Mare	Paint Horse						X	X
10	Unaffected	Mare	Quarter Horse						X	X
15	Unaffected	Gelding	Quarter Horse			X	X			
17	Laminitic	Gelding	Quarter Horse		X			X	X	
4	Laminitic	Gelding	Thoroughbred	X	X		X	X	X	
4	Laminitic	Gelding	Thoroughbred		X			X	X	
19	Laminitic	Gelding	Thoroughbred		X				X	
13	Laminitic	Mare	Quarter Horse	X	X	X		X	X	
7	Laminitic	Gelding	Quarter Horse		X			X	X	X
17	Laminitic	Mare	Quarter Horse	X						X
6	Laminitic	Gelding	Quarter Horse			X				

### Radiographs

**Figure 1:**
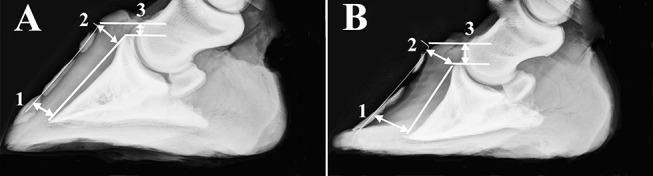
Radiographs of a representative unaffected **(A)** and laminitic hoof **(B)** demonstrating where distances were measured to determine capsular rotation (1-2) and distal descent (3). Specifically, dorsal rotation was quantified by subtracting the distance between the distal inner hoof wall and the third phalanx (1) from the distance between the proximal hoof wall and the third phalanx (2). Sinking was the distance between the proximal portion of the coronary band and the extensor process of the third phalanx (3).

Hooves were cleaned with soap and water and any shoes removed. Lateral digital radiographs were performed immediately after limb collection with a portable radiography unit (70 kVp, 4l mAs, MiKasa X-ray Co, Japan) following placement of a radiopaque wire (length: 76.2 cm; diameter: 0.05 cm) on the dorsal midline of each hoof. A scale bar was included in radiographs to calibrate software tools used to make measures on digital images (Adobe Photoshop, version 5.5, Adobe Systems Inc., Seattle, WA). Dorsal rotation was quantified by subtracting the distance between the distal inner hoof wall and the third phalanx (P3) from the distance between the proximal hoof wall and P3 (Figure 1) (Kummer et al., 2004). Sinking was the distance between the proximal portion of the coronary band and the extensor process of P3.

### Tissue Harvest

Within 2 h of harvest, hooves were cleansed with alternating washes of 10% sodium hypochlorite, sterile water and 0.1% chlorhexidine. With each lateral hoof surface gripped in a vice, two parallel cuts through hoof wall to bone were made 2 cm apart on the dorsal surface from coronary band to toe using a reciprocating saw and sterile blades (DC385, Dewalt®, Towson, MD). The cornified stratum externum and stratum medium between the cuts was elevated with a hoof nipper to expose the stratum internum. A square of approximately 2 × 2 cm was extracted from the midpoint of the dorsal surface with a #20 scalpel blade. The tissue was rinsed with 0.01% chlorhexidine then soaked in phosphate buffered saline (PBS, Hyclone, Logan, UT) containing 1% antibiotic solution (MP Biomedical, Irvine, CA) for 15–30 min at room temperature.

### Cell Isolation and Culture

In a sterile petri dish (Nunc™ IVF petri dish, Thermo Fisher Scientific, Inc., Waltham, MA), tissue was minced into 1 × 1 mm squares using a #10 scalpel blade and then transferred to 50 ml sterile tubes containing collagenase digest composed of 1% bovine serum albumin (BSA) (Sigma-Aldrich, St. Louis, MO) and 0.1% type-1 collagenase (Worthington Biochemical Corporation, Lakewood, NJ) in Dulbecco's modified Eagle's medium with Ham's F12 nutrient mixture (DMEM-Ham's F12, Hyclone). Samples in digest at a 1:2 tissue to digest (v/v) ratio were incubated at 37°C for 2 h with 3-dimensional agitation (5.5 × g, GyroTwister™ GX-1000, LabNet, Inc., Edison, NJ). Subsequently, the digest was passed through a 100 μm followed by a 70 μm filter (Thermo Fisher Scientific) and then centrifuged at 260 × g for 5 min. The stromal vascular fraction (SVF) was resuspended in stromal medium [DMEM-Ham's F12, 1% antibiotic solution (MP Biomedical, Irvine, CA), 10% characterized fetal bovine serum (VWR Life Science, Radnor, PA)] and cultured on 10 cm culture plates (Corning Inc., Corning, NY). Medium was refreshed after 24 h and then every 3 days until 80% cell confluence. For study purposes, seeding density (5 × 10^3^ cells/cm^2^), culture conditions (37°C, 5% CO_2_), and plate confluence (80%) for cell detachment (0.05% trypsin, Hyclone) and passage were conserved throughout unless otherwise indicated.

### Colony Forming Unit Frequency (P0, 2, 5)

Limiting dilution assays were used to determine progenitor cell CFU frequency percentage by established methods (Zhang et al., 2014). Cells were added to a 48-well plate at 5 × 10^3^, 2.5 × 10^3^, 1.25 × 10^3^, 6.25 × 10^2^, 3.12 × 10^2^, and 1.56 × 10^2^ cells/well in individual columns (8 replicates per cell number). After 7 days of culture in stromal medium, wells were rinsed with PBS and fixed with 1% paraformaldehyde (Sigma-Aldrich) in PBS at room temperature for 20 min followed by staining with 0.1% toluidine blue (Sigma-Aldrich) for 1 h and a distilled water rinse. Digital images were obtained with an inverted phase contrast microscope (Olympus® CKX41SF, Japan) instrumented with a digital camera (Olympus DP21, Japan). Positive wells contained 10 or more stained colonies. The CFU frequency was calculated as F = e^−x^, where F = the ratio of negative to total wells, e = natural logarithm constant 2.71 and x = CFU number per well. The CFU frequency was expressed as a percentage (1/CFU frequency×100) (Zhang et al., 2013; Duan and Lopez, 2016).

### Cell Doublings and Doubling Time (P1-3)

Cell expansion was quantified in 12 well plates (Nunclon®, Sigma-Aldrich) as previously described (Zhang et al., 2013). Cell numbers were evaluated after 2, 4, and 6 days of culture using trypan blue exclusion and a hemocytometer. Cell doublings (CD) and doubling time (DT) were calculated as CD = ln(N_f_ /N_i_)/ln(2) and DT = CT/CD where CT = culture time, N_f_ = final cell number, and N_i_ = initial cell number.

### Multi-Lineage Differentiation (P1)

Adipogenic, osteogenic and neurogenic differentiation was confirmed in cell isolates from U and L hooves cultured in 6 well plates (Thermo Fisher Scientific). Cells were cultured in stromal medium for 5 days to about 80% confluence followed by culture in induction medium. For adipogenesis, cells were cultured in adipogenic induction medium [DMEM-Ham's F12, 3% FBS, 1% antibiotic solution, biotin (33 mmol/L), pantothenate (17 mmol/L), insulin (1 mmol/L), dexamethasone (1 mmol/L), isobutylmethylxanthine (IBMX, 0.5 mmol/L), rosiglitazone (5 mmol/L) (TZD, AK Scientific, Union City, CA), 5% rabbit serum (Invitrogen Corporation, Carlsbad, CA)] for 3 days followed by adipogenic maintenance medium (adipogenic medium without IBMX or rosiglitazone) for 2 more days. To confirm lipid accumulation, cells were fixed for 2 h in 4% paraformaldehyde at room temperature and then stained with oil red O. To assess osteogenesis, cells were cultured in osteogenic induction medium [DMEM-Ham's F12, 10% FBS, 1% antibiotic solution, β-glycerophosphate (1 mol/L), dexamethasone (20 nmol/L), sodium 2-phosphate ascorbate (50 mg/mL)] for 14 days and then fixed with 70% cold ethanol for 2 h. Calcium in colonies was stained with 2% alizarin red in distilled water (pH 4.2) for 15 min at room temperature and then rinsed with distilled water (Vidal et al., 2007). Cells were also cultured in neurogenic pre-induction medium (DMEM, 10% FBS, 1 mM 2-mercaptoethanol) for 2 days followed by neurogenic induction medium (DMEM, 5.5 mM glucose, 120 μM indomethacin, 10% FBS, 3 μg/ml insulin, 300 μM IBMX) for 3 days. They were fixed with 1% paraformaldehyde overnight and then blocked with 10% goat serum for 1 h at 37°C. Cells were incubated with a mouse anti-microtubule-associated protein (MAP2) primary antibody (1:250) for 2 h at 37°C. They were then incubated with anti-mouse immunoglobulin G (IgG) secondary antibody labeled with Alexa Fluor 488 (1:1,000) (Table 2) for 1 h at room temperature. Controls included cells cultured in stromal medium and induced cells incubated with secondary antibody alone. Nuclei were stained with 4, 6-diamidino-2-phenylindole (DAPI, 1 μg/ml) for 10 min followed by a distilled water rinse. Photomicrographs were obtained with a camera (DFC480, Leica Microsystems, Germany) on a fluorescent microscope (DM 4500b, Leica Microsystems).

**Table 2:** Antibody information.

**Antibody**	**Label**	**Description**	**Cross/ Reactivity**	**Host**	**Manufacturer**	**Catalog #**.	**Diluent**
CD29	N/A	Integrin β-1	Human, Mouse, Rat, Dog, Chicken	Mouse	BD Biosciences	BS610468	PBS
CD44	FITC	Hyaluronic Acid Receptor	Human, Mouse	Rat	EBiosciences	11044182	PBS
CD105	PE	Endoglin	Human, Pig	Mouse	Abcam Inc	Ab69772	PBS
K14	N/A	Keratin 14	Human, Rat, Human	Mouse	Abcam Inc	Ab7800	PBS
K15	N/A	Keratin 15	Human, Cow, Rat, Mouse	Mouse	Abcam Inc	Ab80522	PBS
K19 MAP2 IgG IgG IgG IgG DSG1 DSG3	N/A N/A FITC Alexa Fluor 633 Alexa Fluor 488 Alexa Fluor 594 N/A N/A	Keratin 19 Microtubule Protein Anti-mouse Anti-mouse Anti-mouse Anti-mouse Desmoglein-1 Desmoglein-3	Human Mouse, Rat Mouse Mouse Mouse Mouse Human Human	Mouse Mouse Goat Goat Donkey Goat Mouse Mouse	Abcam Inc FisherScientific Sigma-Alorich FisherScientific FisherScientific FisherScientific Invitrogen Invitrogen	Ab7754 13-1500 F0257 A-21052 A-21202 R37121 32-6000 32-6300	PBS PBS PBS PBS PBS PBS TBS TBS

### Immunohistochemistry (IHC), Immunocytochemistry (ICC) (P1, 3)

IHC (fluorescent)-Fresh tissue was embedded in optimal cutting temperature compound (OCT, Sakura Finetek Inc., Torrance, CA), solidified at −80°C, sectioned (5 μm) with a cryostat (Leica® CM1850, Sarasota, FL), and applied to slides (poly L-lysine coated, Sigma-Aldrich). Sections were blocked with 10% goat serum (Abcam Inc., Cambridge, MA) in PBS for 1 h at room temperature after rehydration in PBS for 10 min. Slides were incubated with individual primary antibodies (CD29, CD44, K14, K19) (Table 2) diluted in tris-buffered saline (TBS, 1:200) at 37°C for 2 h, rinsed with PBS, incubated with anti-mouse IgG-Alexa Fluor 594 at 37°C for 1 h in darkness, and then rinsed with PBS again. Nuclei were stained with Hoechst's dye (Biotium, Hayward, CA), for 10 min at room temperature in darkness. Digital images were obtained with a fluorescent microscope (DM 4500b, Leica) equipped with a digital camera (DFC 480, Leica). Negative controls for unlabeled antibodies included sections incubated with secondary antibody alone. Despite the fact that CD44 had a conjugated FITC label, sections labeled with CD44 were incubated with the same secondary antibody as unconjugated antibodies for consistency. The label does not interfere with the reaction between the primary and secondary antibodies.

IHC (chromogen)-Formalin fixed sections of laminae (1 × 0.5 × 0.5 cm) were paraffin embedded and sectioned (5 μm). Antigen retrieval was performed by incubating in citrate buffer (pH 6) for 30 min at 80°C. Sections were rinsed in PBS and endogenous peroxidase was blocked by incubation in 3% H_2_O_2_ for 30 min at room temperature. Non-specific binding of antibodies was blocked by incubation with 1% BSA (Sigma-Aldrich) and 1% pre-immune serum (Abcam) in PBS for 1 h at 37°C. Sections were then immunostained with murine anti-human antibodies against K14 or K15 (Table 2) overnight at room temperature. After rinsing in PBS, sections were incubated with horse radish peroxidase (HRP) conjugated anti-mouse IgG (DAKO EnVision, Dako, Carpinteria, CA) for 1 h at room temperature. Bound antibodies were then exposed with diaminobenzidine/H_2_O_2_ staining for 3 min at room temperature. Slides were counterstained with hematoxylin and digital images captured with a light microscope (DM5000B, Leica) instrumented with a camera (DFC 480, Leica). Sections treated with secondary antibody alone were included as negative controls.

### ICC

Cells were seeded on eight well multi-chamber slides (7 × 10^3^ cells/well, Thermo Fisher Scientific). At approximately 70% confluence, cells were fixed in 4% paraformaldehyde. They were then permeabilized with 1.0% Triton X-100 (Sigma-Aldrich) in PBS for 10 min at room temperature before blocking with 10% goat serum (Abcam) and 2% BSA (Sigma-Aldrich) in 1.0% Triton X-100. Cells were incubated with primary antibodies against desmoglein (DSG) 1, DSG3, K15, and K19 (Table 2) diluted 1:200 in the blocking solution above for 2 h at 37°C followed by goat anti-mouse IgG-Alexa Fluor 594 (1:1,000, Table 2) at room temperature for 2 h in darkness. Nuclei were stained with 4, 6-diamidino-2-phenylindole (DAPI). Photomicrographs were obtained with a fluorescent microscope (DM 4500b, Leica) equipped with a digital camera (DFC 480, Leica). Negative control samples consisted of cells incubated with secondary antibody alone.

### Flow Cytometry (P0, 2, 5), Neurogenesis (P1)

Antibodies against K14, K15, K19, CD29, CD105, CD44 and MAP2 (Table 2) were used to label cells for flow cytometry. With the exception of MAP2, antibodies were conjugated with FITC (FluoroTag™ FITC conjugation kit, Sigma-Aldrich). Cell aliquots (10^5^ cells) were suspended in 800 μl of 1% BSA and 0.2% sodium azide in PBS containing 2 μl (200 ug/ml) of labeled antibody, and the mixture was incubated at room temperature for 30 min in darkness. Incubation with keratin antibodies was performed after cell permeabilization (Cytofix/Cytoperm™ kit, BD Biosciences, San Jose, CA). Following incubation with antibodies, cells were rinsed with PBS and fixed with 4% neutral buffered formalin. For MAP2 detection, cells were additionally incubated with anti-mouse IgG-Alexa Fluor 633 (Thermo Fisher Scientific). Cell fluorescence was quantified using a FACSCalibur flow cytometer (BD Biosciences, San Jose, CA) and CellQuest graphics software (BD Biosciences). Unlabeled cells and isotype controls were included for all assays.

### RT-PCR (P0, 2, 5)

Equine specific primers were designed for CD44, CD105, K14, K15, OCT4 and SOX2 (Table 3) using publicly available software (Primer-BLAST) and according to standard criteria established by the National Center for Biotechnology. Cells were detached from culture ware with 0.05% trypsin (Hyclone), rinsed with PBS and centrifuged at 260 × g for 5 min. Total mRNA was isolated (RNAqueous Micro Kit Ambion, Inc., Austin, TX). Potential DNA contamination was removed by DNase I (Turbo DNA-free, Ambion, Inc., Austin, TX) digestion. Samples were reverse transcribed into complementary DNA (cDNA) using oligo (dT) primers and Moloney Murine Leukemia Virus (M-MLV) reverse transcriptase (Sigma-Aldrich). Target gene mRNA levels were quantified with qRT-PCR using SYBR Green technology (Thermo Fisher Scientific) and an MJ Research Chromo4 Detector (Bio-Rad Laboratories, Hercules, CA). Amplicons were sequenced to confirm target sequence amplification. The ΔCt values were determined relative to the reference gene glyceraldehyde 3-phosphate dehydrogenase (GAPDH).

**Table 3:** Primer sequences.

**Gene**	**Forward**	**Reverse**	**Accession No**.
GAPDH	CAA TGA CCC CTT CAT TGA CC	GAA GAT GGT GAT GGC CTT TC	NM_001163856
SOX-2	CAG CTC GCA GAC CTA CAT GA	TGG AGTG GGA GGA AGA GGT A	XM_023623361
OCT4	TCG TTG CGA ATA GTC ACT GC	AGT GAGA GGC AAC CTG GAG A	XM_023624232
CD29	CCA TTG TTC ACG TTG TGG AG	TTG GCA AAT TCC CTT CTG TC	XM_023631884
CD44	CAG CAC CCC TGC GGA TGA CG	TGG TCT TGG GTG GGG CGA GT	XM_023653788
CD105	CCC CAA GAG TCA ACA CCA CT	GTT CGA GAC TGC AGG AGG AC	XM_003364144
K14	TAC GAG ACG GAG CTG AAC CT	TGG CCT CTC AGG CTA TTC AT	XM_001346198
K15	GTG GCT TTG GTG ACT TTG GT	GTC TCG GAT CTT CAC CTC CA	XM_005597407

### *In vivo* Extracellular Matrix Production (P3)

Scaffold carriers composed of polyethylene glycol/poly-L-lactic acid (60:40, GA) and tricalcium phosphate/hydroxyapatite (40:60, HT) were fabricated by thermally induced phase separation as previously described (Smoak et al., 2015). Equine laminar progenitor cells (P1) from U hooves were cryopreserved in liquid nitrogen in custom medium (10% dimethyl sulfoxide, 10% DMEM/F-12, 80% FBS) after being gradually cooled to −80°C (−1°C/minute, CoolCell®, BioCision, Larkspur, CA). They were then culture expanded to P3 and loaded (6.3 × 10^3^ cell/mm^3^) onto scaffolds (diameter: 10 mm; depth: 3 mm) for 2 h at 70 rpm stirring in a spinner flask (Bellco® Biotechnology, Newark, NJ) containing 120 ml of serum-free stromal medium. Cells that remained in the medium were quantified to estimate the number of cells on the scaffolds. Scaffolds without cells were treated identically to those with cells prior to implantation. One scaffold with or without cells divided into six equal pieces was surgically implanted in the dorsal subcutaneous tissues of each of four male athymic mice (nu/nu, Charles River Laboratories, Wilmington, MA). Specifically, six pieces of an HT cell-scaffold construct was implanted in one mouse, six pieces of a GA cell-scaffold construct in another, six pieces of an HT scaffold in another, and one mouse received six pieces of a GA scaffold.

For the implantation procedure, mice were premedicated with glycopyrrolate (0.02 mg/kg) and butorphanol (0.5 mg/kg) and subsequently anesthetized with isoflurane on oxygen delivered via a Baine circuit and mask. Following aseptic preparation, 6 skin incisions (5 mm) were created equidistantly along the dorsum extending from the scapula to the sacrum, approximately 1 cm lateral to each side of the spine, and scaffolds were placed after minimal blunt dissection. Subcutaneous tissues were closed with monocryl (Monocryl®, Cornelia, GA) and skin apposed with tissue glue (Sigma-Aldrich). Nine weeks after implantation, explants were fixed with 2.5% glutaradehyde in 0.1 M sodium carcodylate buffer, post-fixed with 0.1% osmium tetroxide, and dehydrated in a series of ethanol-distilled water solutions. After critical point drying, constructs were sputter coated with gold and imaged with a scanning electron microscope at 15 kVP (FEI Quanta 200, Netherlands) (Xie et al., 2013).

### Transmission Electron Microscopy (P1)

Cells from U and L hooves were detached at 80% confluence and centrifuged at 260 × g for 5 min at room temperature. Pellets (3 × 10^6^ cells/pellet) were fixed with 1.6% paraformaldehyde, 2.5% glutaraldehyde, 0.03% CaCl_2_ in 0.05 M cacodylate buffer (pH 7.4), washed in 0.1 M cacodylate buffer supplemented with 5% sucrose and post fixed in 2% osmium tetroxide for 1 h. They were washed with distilled water and incubated in a 1% water solution of tannic acid for 1 h. Following dehydration with propylene oxide in ascending ethanol concentrations, pellets were embedded in Epon-Araldite (EMS, Hatfield, PA). Blocks were sectioned (80–90 nm, Ultratome Leica EM UC7, Germany) and stained with 2% uranyl acetate in maleate buffer and lead citrate. Images were generated with a digital camera (Hamamatsu ORCA-HR, Japan) on an electron microscope (JEOL JEM 1011, Japan). All reagents for electron microscopy were from the same source (Electron Microscope Sciences, Hatfield, PA).

## Results

### Radiographs

Capsular rotation was 1.0 ± 1.4 mm (mean ± SEM) and 3.7 ± 1.7 mm, and distal descent 9.0 ± 0.8 mm and 10.6 ± 3.7 mm in U and L hooves, respectively (Figure 1).

### Cell Isolation and Culture

**Figure 2:**
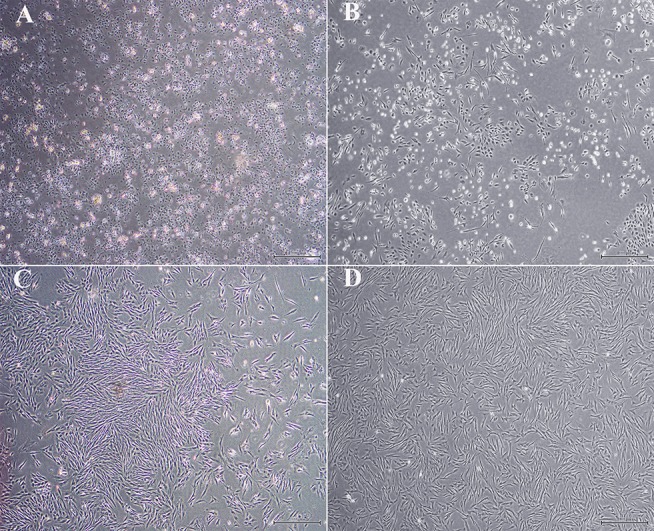
Light photomicrographs of equine hoof progenitor cells from unaffected **(A,C)** and laminitic hooves **(B,D)** after 2 **(A,B)** and 7 days **(C,D)** of culture. Bar = 500 μm.

Cell yield was 1.34 × 10^7^ ± 1.25 × 10^6^ and 1.70 × 10^7^ ± 3.05 × 10^6^ nucleated cells/g from U and L hoof tissue, respectively. Primary cell isolates from all hooves exhibited a rhomboid shape after 2 days of culture (Figures 2A,B). After 1 week's culture, most cells had a typical spindle shape consistent with progenitor cell morphology (Figures 2C,D).

### Colony Forming Unit Frequency (P0, 2, 5)

**Figure 3:**
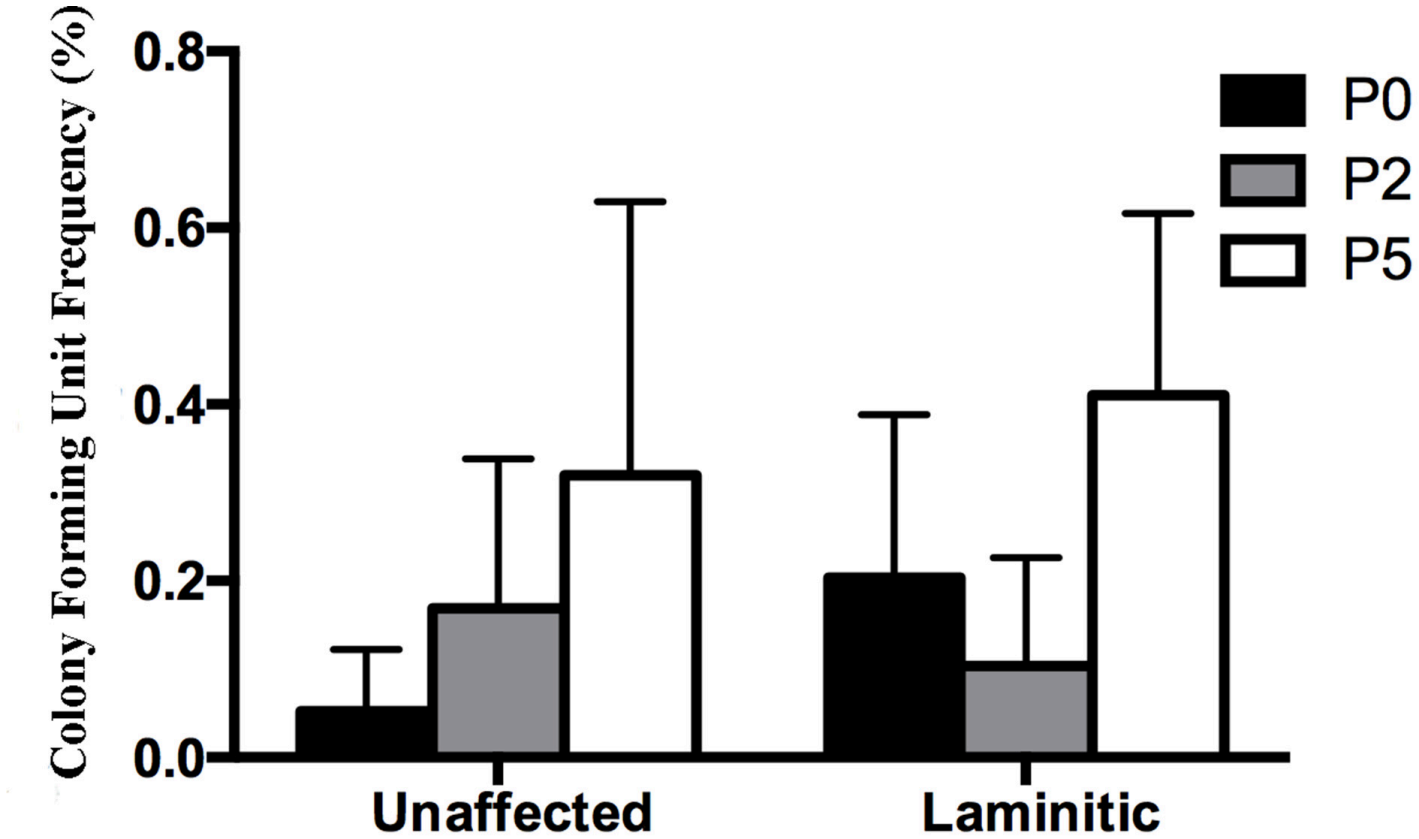
Colony forming unit frequency percentages (mean ± SEM) for P0, 2 and 5 progenitor cells from unaffected and laminitic hooves.

The CFU frequency percentages tended to increase with increasing passage (Figure 3). They were 0.05 ± 0.04%, 0.17 ± 0.07% and 0.32 ± 0.31% for passages P0, 2 and 5, respectively, for progenitor cells from U hooves. For the same cell passages from L hooves, CFU frequency percentages were 0.20 ± 0.09%, 0.10 ± 0.06% and 0.41 ± 0.21%.

### Cell Doublings and Doubling Time (P1-3)

**Figure 4:**
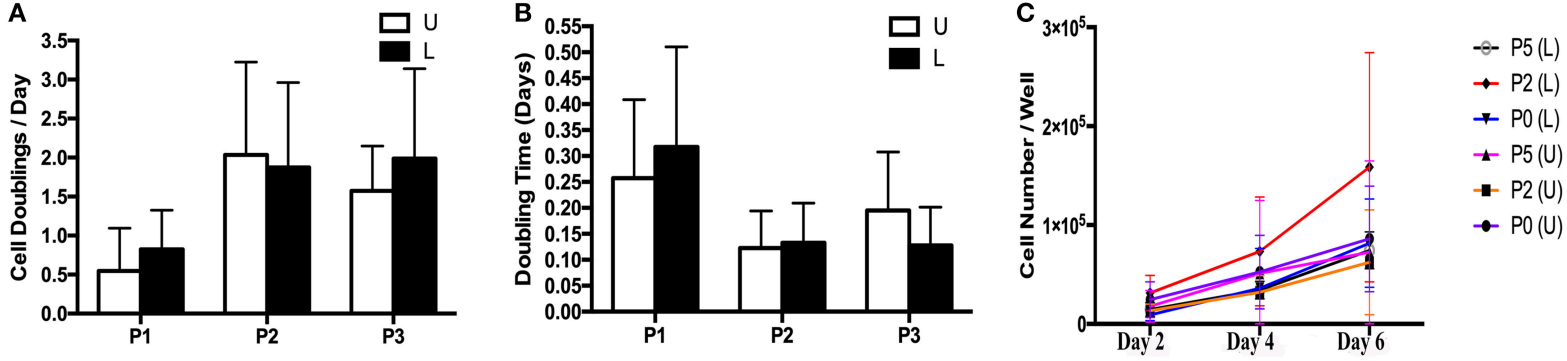
Cell doublings **(A)**, doubling times **(B)** and growth curve **(C)** (mean ± SEM) for P1-3 progenitor cells from unaffected (U) and laminitic (L) hooves.

The rate of *in vitro* expansion tended to increase with passage (Figures 4A–C). There was minimal variation in cell expansion between days for each sample, so data from all days for each sample were combined within passages for purposes of calculating mean and standard error. Cell doubling values for P1, P2 and P3 for cells from U hooves were 0.55 ± 0.65, 2.04 ± 1.20 and 1.58 ± 0.57 vs. 0.85 ± 0.50, 1.88 ± 1.10 and 1.99 ± 1.20 for the same passages from L hooves (Figure 4A). Doubling times for P1, P2 and P3 cells from U hooves were 0.26 ± 0.15, 0.12 ± 0.07 and 0.20 ± 0.11 days/CD vs. 0.32 ± 0.19, 0.13 ± 0.07 and 0.12 ± 0.07 days/CD from L hooves (Figure 4B).

### Multi-Lineage Differentiation (P1)

**Figure 5:**
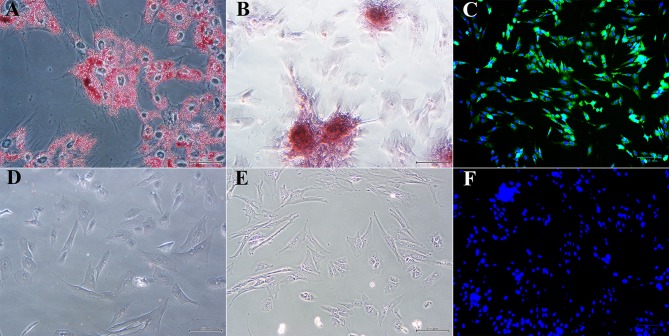
Photomicrographs of P1 laminar progenitor cells cultured in adipogenic **(A)**, osteogenic **(B)**, neurogenic **(C)** or stromal **(D-F)** medium and stained with oil red O **(A,D)**, alizarin red **(B,E)** or anti-map2, anti-mouse IgG-488 (green, **C,F**). DAPI nuclear stain (blue, **C,F**). Magnification = 20X, Bar = 100 μm.

Passage 1 progenitor cells from U and L hooves all displayed characteristic adipocytic (Figure 5A), osteoblastic (Figure 5B) and neurocytic (Figure 5C) differentiation following culture in differentiation medium based on histochemical or immunocytochemical staining. Cells cultured in stromal medium for identical time periods did not differentiate (Figures 5D–F).

### Immunohistochemistry (IHC), Immunocytochemistry (ICC) (P1, 3)

**Figure 6:**
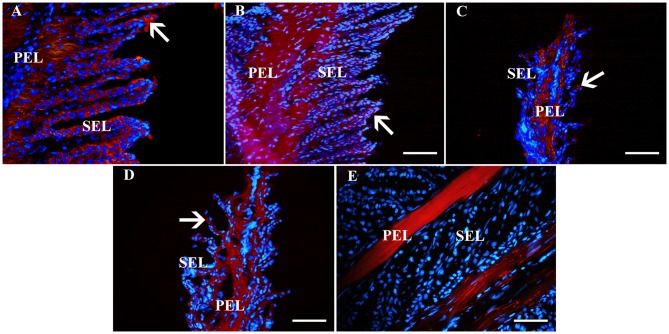
Fluorescent photomicrographs of primary and secondary epidermal lamellae from an unaffected hoof labeled with antibodies (red) against K14 **(A)**, K19 **(B)**, CD44 **(C)**, CD29 **(D)** or no antibodies **(E)**. Hoechst nuclear dye (blue) and Hoechst nuclear dye (blue). PEL, primary epidermal laminae; SEL, secondary epidermal laminae; Arrows = stained cells; Magnification = 40X, Bar = 50 μm.

**Figure 7:**
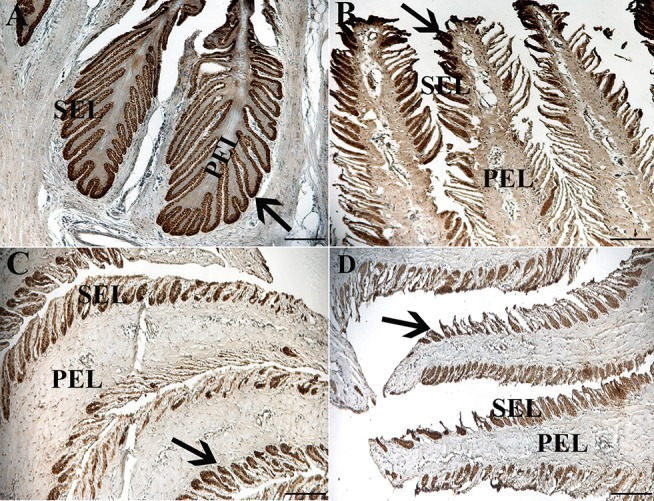
Light photomicrographs of lamellae from unaffected **(A,B)** and laminitic **(C,D)** hooves labeled (brown) with antibodies against K14 **(A,C)** and K15 **(B,D)**. PEL, primary epidermal laminae; SEL, secondary epidermal laminae; Arrow = labeled cells; Magnification = 20X **(A)**, Bar = 100 μm; Magnification = 40X **(B–D)**, Bar = 50 μm.

**Figure 8:**
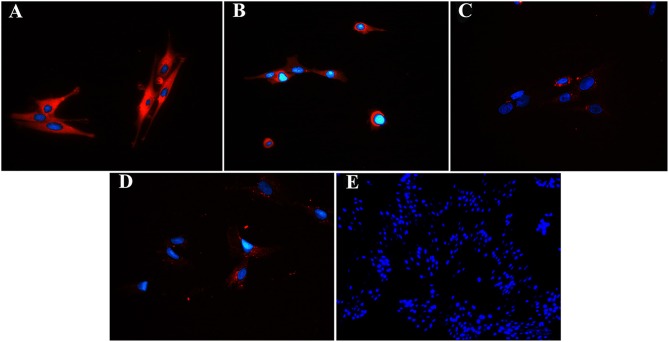
Fluorescent photomicrographs of cultured progenitor cells (P3) from unaffected **(A,B)** and laminitic **(C,D)** hooves labeled (red) with anti- DSG1 **(A,C)**, DSG3 **(B,D)** antibodies or no antibodies **(E)**. DAPI nuclear stain (blue); Magnification = 40X **(A–D)**; 20X **(E)**.

**Figure 9:**
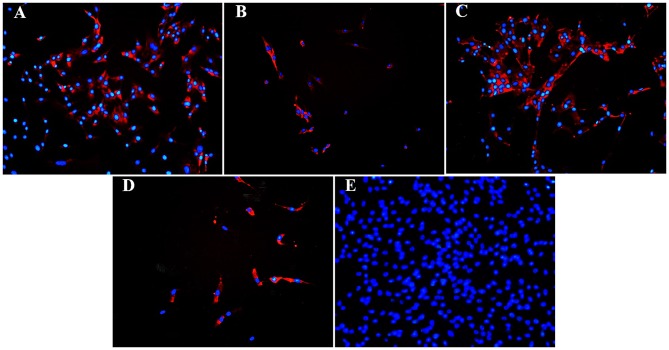
Fluorescent photomicrographs of cultured hoof progenitor cells (P1) from unaffected **(A,B)** and laminitic **(C,D)** hooves after labeling (red) with antibodies against K15 **(A,C)**, K19 **(B,D)** or no antibodies **(E)**. DAPI nuclear stain (blue); Magnification = 20X.

Fluorescent (Figure 6) and chromogen (Figure 7) immunohistochemical labeling confirmed the presence of CD44, CD29, K14, K15 and K19 in the stratum internum of both U and L hoof tissue. Based on immunocytochemical staining, cultured cells from U and L hooves expressed DSG1 and DSG3 (P3, Figure 8) as well as K15 and K19 (P1, Figure 9).

### Flow Cytometry (P0, 2, 5), Neurogenesis (P1)

**Figure 10:**
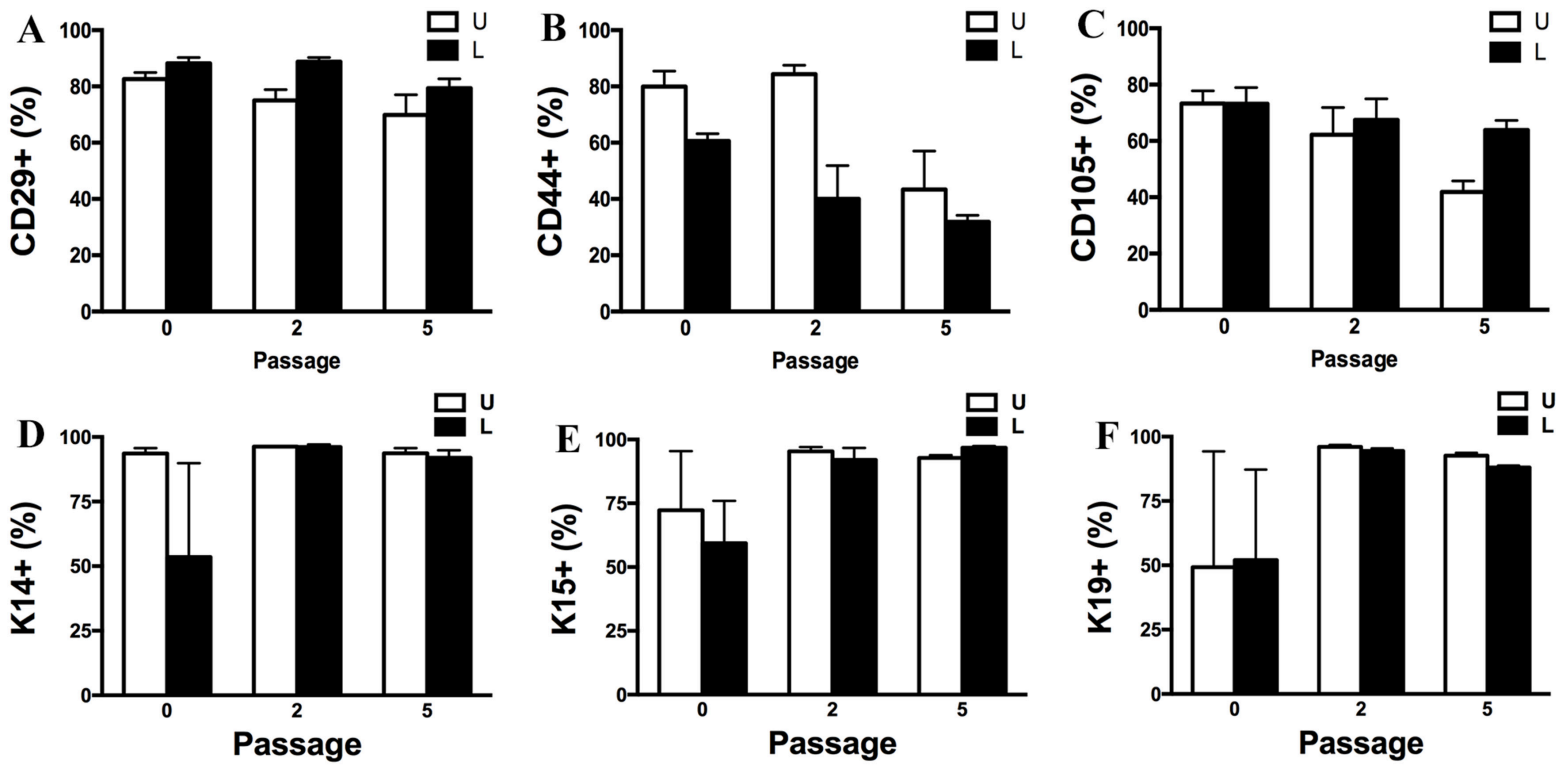
Percentage (mean ± SEM) of CD29^+^ **(A)**, CD44^+^ **(B)**, CD105^+^ **(C)**, K14^+^ **(D)**, K15^+^ **(E)** and K19^+^ **(F)** P0, 2 and 5 progenitor cells from unaffected (U) and laminitic (L) hooves based on flow cytometry.

**Figure 11:**
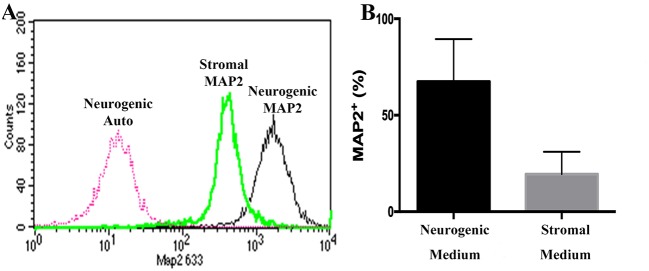
Flow cytometry histogram showing MAP2 labeling of P1 equine hoof progenitor cells from an unaffected hoof cultured in stromal (Stromal MAP2) or neurogenic medium (Neurogenic MAP2), and autofluorescence of unlabelled cells cultured in neurogenic medium (Neurogenic Auto, **A**). Percentage (mean ± SEM) of P1 hoof progenitor cells from unaffected hooves expressing MAP2 after culture in stromal (Stromal) or neurogenic (Neurogenic) induction medium **(B)**.

Passage P0, 2 and 5 cultured progenitor cells from U and L hooves expressed CD29, CD44, CD105, K14, K15, and K19 (Figure 10). The percentage of cells expressing CD105 or CD44 (Figures 10B,C) tended to decrease with passage while the percentage expressing CD29 (Figure 10A) remained fairly constant across passages. The majority of P2 and 5 from both hoof conditions expressed K14, K15 and K19 (>90%) (Figures 10D–F). Approximately 70% of P1 cells cultured in neurogenic induction medium expressed MAP2 compared to 18% of cells cultured in stromal medium (Figure 11).

### RT- PCR (P0, 2, 5)

**Figure 12:**
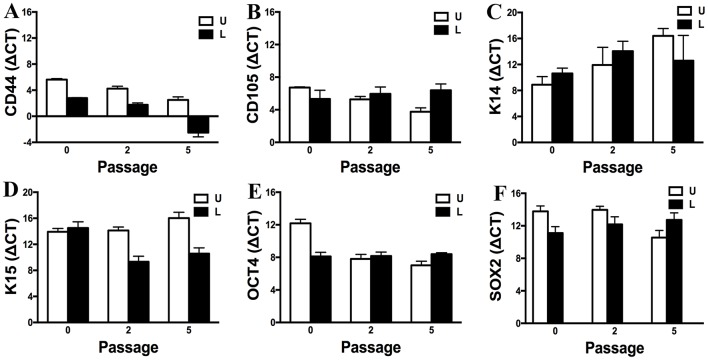
Expression levels (ΔCT, mean ± SEM) of CD44 **(A)**, CD105 **(B)**, K14 **(C)**, K15 **(D)**, OCT4 **(E)** and SOX2 **(F)** in progenitor cells from unaffected (U) and laminitic (L) hooves at the indicated passages. Reference gene: GAPDH; SOX2 = SRY (sex determining region Y)-box 2; OCT4 = octamer-binding transcription factor 4.

Gene expression of CD44, CD105, K14, K15, OCT4, and SOX2 by P0, 2 and 5 progenitor cells from U and L hooves was confirmed (Figure 12). Gene expression was relatively similar between conditions and stable across passages. Exceptions included an apparent decrease in K14 expression with increasing passage in cells from U hooves and an increase in CD44 expression with increasing passage in cells from L hooves. The stability of the reference gene, GAPDH, was confirmed by a CT value of 17.71 ± 0.20 for all samples.

### *In vivo* Extracellular Matrix Production (P3)

**Figure 13:**
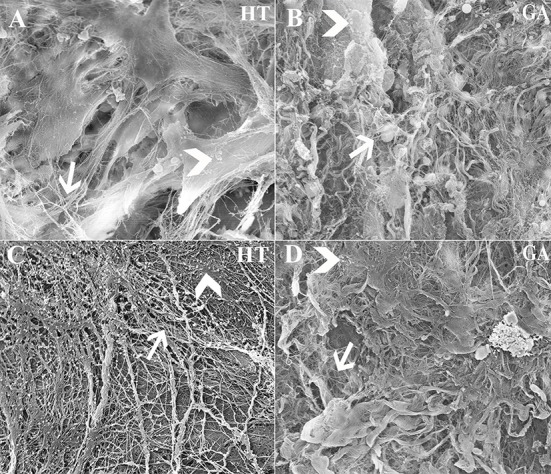
Scanning electrophotomicrographs of scaffold constructs with **(A,B)** and without **(C,D)** P3 equine hoof progenitor cells from an unaffected hoof 9 weeks after subcutaneous implantation in athymic mice. Arrow = collagen fibrils; Arrow head = organized extracellular matrix; Magnification = 6,000X **(A,C)**, 3000X **(B,D)**.

Approximately 5.60 × 10^3^ cells/mm^3^ were loaded onto each scaffold. Abundant collagen fibrils and organized ECM was apparent on scaffolds containing cells (Figures 13A,B), while those without cells contained fewer collagen fibrils and little organized ECM (Figures 13C,D).

### Transmission Electron Microscopy (P1)

**Figure 14:**
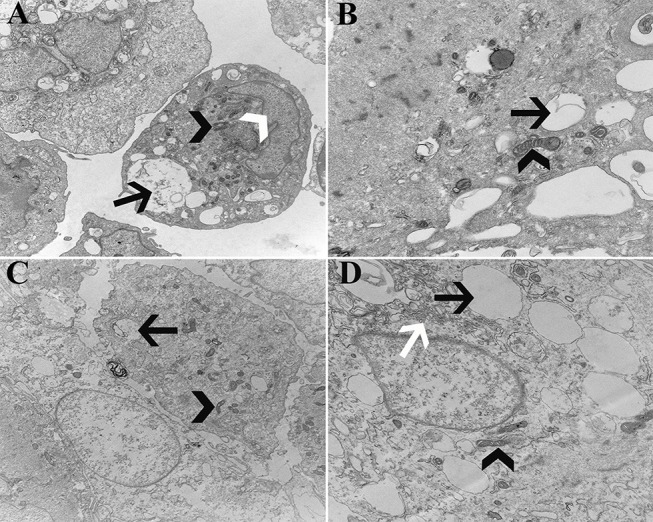
Transmission electromicrographs of progenitor cells (P1) from unaffected **(A,B)** and laminitic **(C,D)** hooves. Black arrow = vacuole; White arrow head = nucleus; Black arrow head = mitochondria; White arrow = rough endoplasmic reticulum; Magnification = 10,000X **(A,C)**, 20,000X **(B,D)**.

Cell ultrastructure was similar between cells from U and L hooves (Figure 14). Large, irregular, eccentric nuclei, elongated mitochondria, rough endoplasmic reticulum surrounded by cisternae, and randomly distributed cytoplasmic vacuoles were present in all cells evaluated.

## Discussion

The work in this study establishes a reproducible mechanism to isolate and culture progenitor cells from unaffected and chronically inflamed ectodermal-mesodermal tissue of the equine hoof, a tissue interface similar to the human and mouse periodontal membrane and the human fingernail (Leung et al., 2014; Maiolino et al., 2016). *In vitro* culture of adult progenitor cells provides a mechanism to evaluate cell behavior under distinct conditions (Gronthos et al., 2000; Caplan, 2007; Paschos et al., 2015; Khojasteh et al., 2017) to assess cell alterations mediated by chronic inflammation or disease. The methods will support cell and tissue culture for investigation of therapies to prevent or address pathogenic processes in ectodermal-mesodermal tissue. This work establishes a unique and important model to study progenitor cells in the distinct ectodermal-mesodermal niche, a site of important health challenges in both veterinary and human patients (Gross et al., 2005; Martins et al., 2016).

Plastic adherence of the cells in this study is consistent with cell immaturity and multi-lineage differentiation confirms plasticity (Dominici et al., 2006; Bourzac et al., 2010; Fulber et al., 2016). The immature status of the cell isolates is supported by relatively stable expression of genes associated with progenitor cell self-renewal and proliferation, OCT4 and SOX2 (Bourguignon et al., 2012). Progenitor cells from both inflamed and unaffected tissues showed similar *in vitro* behavior as equine progenitor cells from normal adult tissues (Koerner et al., 2006; Violini et al., 2009; Bourzac et al., 2010; Mccarthy et al., 2012), and characteristics appeared to be maintained through numerous passages (Jiang et al., 2017). Notably, mesodermal and ectodermal cell differentiation suggests the presence of cells from different lineages, cells capable of transdifferentiation, or, most likely, both given the heterogeneous composition of the isolates. Somewhat consistent expression of both mesodermal, CD44 and CD105, and ectodermal, K15 and K14, progenitor cell genes up to five passages further confirms the likely presence of multiple cell lineages and/or transitional cells and indicates that the unique cell identities are well maintained in culture (Ranera et al., 2011; Troy et al., 2011; Hayashi et al., 2016). *In vitro* expansion rates in this study were slightly higher than those reported for bone marrow, adipose and umbilical cord derived equine MSCs of about 1 day/CD (Vidal et al., 2012) and human epidermal keratinocytes of about 0.7 days/CD (Sun and Green, 1976). Distinct culture conditions and natural variation typically contribute to differences in expansion behavior among studies (Gerhard et al., 2004; Sotiropoulou et al., 2006). The CFU frequency percentages in this study are comparable to those from mesodermal and ectodermal equine progenitor cells of about 0.6 and 1.5%, respectively (Gargett et al., 2009; Corradetti et al., 2011). Taken together, these results confirm the immaturity and diversity of the heterogeneous primary cell isolates.

The presence of mesodermal and ectodermal proteins in hoof tissue and within heterogeneous cell isolates *in vitro* is important to translate information between *in vitro* and *in vivo* conditions (Michel et al., 1996; Ponta et al., 2003; Fonsatti and Maio, 2004; Schaffler and Buchler, 2007; De Mattos Carvalho et al., 2009; Abbas et al., 2011; Halfon et al., 2011; Bose et al., 2013; Liao et al., 2014). A prominent goal of this series of investigations was to validate parallel protein expression in cultured primary cell isolates and tissue. Despite a plethora of methods to culture progenitor cells from numerous tissues of most species, localization of progenitor cell antigens *in situ* and *in vitro* is rare (Galantino-Homer et al., 2014; Ghasemi, 2017). The fact that the cell isolates retained their characteristics in culture based on flow cytometry results is especially vital to the validity of the culture model. Results of this study are limited to cells that have been passaged beyond the initial stromal vascular fraction isolate. Culture of cells under ideal conditions can potentially mask natural attributes and behaviors, a recognized limitation of cell culture and impetus to maintain culture conditions that replicate distinct natural states (Duval et al., 2017).

The ability of cells within heterogeneous primary cell isolates to mature into distinct lineages in this study is consistent with current knowledge surrounding cell plasticity, especially in tissue interfaces (Pearton et al., 2005; Medvedev et al., 2010; Rohani et al., 2011). As indicated above, it is likely that the junction between epidermal and dermal tissues contains progenitor cells from ectodermal and mesodermal embryonic layers, and, potentially, transitional cells that have characteristics of both tissue types permitting ready transdifferentiation (Phinney and Prockop, 2007). Additional work is needed to isolate and characterize specific cell immunophenotypes from heterogeneous primary cell isolates.

Tight cell junctions that stabilize the ectodermal-mesodermal tissue interface are composed of a variety of proteins like desmogleins, cadherins, laminins and E-selectins, among others (Leise et al., 2011). Desmogleins are major proteins of desmosomes, cell to cell junctions (Pollitt, 2016), and hemidesmosomes, cell to tissue or ECM junctions. Loss of desmoglein integrity or diminished production is associated with sloughing of the epidermis (Miragliotta et al., 2006; Thomason et al., 2010; Amagai and Stanley, 2012; Saito et al., 2012) and abnormal tissue production (Walko et al., 2015; Najor and Green, 2018). There are fewer hemidesmosomes along the basement membrane between the ectodermal and dermal layers in inflamed vs. normal equine hoof tissue (Nourian et al., 2007). Future studies using cell culture may further confirm the potential for disruption of desmoglein function or production by inflammation.

The cells in this study shared some ultrastructural features with equine and human adult multipotent cells including polymorphic nuclei, well-developed dilated cisternae of rough endoplasmic reticulum, intracellular vacuoles and elongated mitochondria (Horstmann et al., 2002; Pascucci et al., 2010; Rodrigues et al., 2010; Alipour et al., 2015; Miko et al., 2015). High metabolic activity typical of immature cells is suggested by elongated mitochondria (Campello and Scorrano, 2010). Intracellular vacuoles are associated with protein recycling (Hwang et al., 2014; Zheng et al., 2014), and they are thought to be filled with proteinaceous material destined for secretion in human adult stem cells from bone marrow (Colter et al., 2001; Raimondo et al., 2006). There are also reports of small cytoplasmic vacuoles in Merkel cells within canine whisker pad basal epidermis (Hashimoto, 1972; Ramirez et al., 2015). The ultrastructure of the cells reported here is consistent with high metabolic activity of progenitor cells and a secretory function typically associated with keratinizing cells (Gallucci et al., 1979). The results are not consistent with any one cell type and likely reflect the heterogeneous nature of the cell isolates.

Abnormal tissue formation in inflamed ectodermal-mesodermal interface tissues has been reported to be the result of altered maturation (Eastham et al., 2007; Rucker, 2010; Galantino-Homer et al., 2014; Lamouille et al., 2014; Bailey, 2015; Tomasello et al., 2017). As such, appropriate cell direction is essential for stem cell transplantation therapies to drive restorative tissue formation (Shokrgozar et al., 2012; Takahashi et al., 2013). The present study was not designed to assess treatment outcomes in contrast to clinical reports of stem cell therapies (Peroni, 2013; Morrison et al., 2014) or to compare characteristics of cells from normal and inflamed tissue, but to establish a model of cell culture that accommodates cells from both tissue conditions. Robust, organized ECM formation by hoof progenitor cells on scaffold carriers confirms the translational potential of the cell isolates from this study (Rustad et al., 2012; Duan et al., 2018). However, future work is necessary to formulate scaffold carriers to direct hoof progenitor cell differentiation *in vivo* or support hoof tissue formation *in vitro* to create implantable graft tissue that guides native progenitor cells (Sundelacruz and Kaplan, 2009; Qi et al., 2014). Evaluation of the broad cell signaling pathways represented by OCT4 and SOX2 in primary cell isolates cultured under conditions that replicate native tissue may be natural starting points for study of mechanisms to modulate cell behaviors in tissue constructs (Zhou et al., 2013; Amini et al., 2014; Matic et al., 2016; Sardarabadi et al., 2016). In the future, *de novo* tissue formation with progenitor cells may provide a new therapeutic option for chronic conditions of the ectodermal-mesodermal interface.

The authors recognize several limitations of this preliminary study to establish isolation and culture of progenitor cell isolates from an ectodermal-mesodermal interface. The characteristics of a cell population reflect but are not identical to those of individual cells (Altschuler and Wu, 2010). As such, work is necessary to isolate and evaluate differences among distinct immunophenotypes within the mixed (heterogeneous) populations included in this study. Also, as pointed out above, the initial 2D cell culture in mesodermal basal medium as well as the isolation process very likely selected for distinct progenitor cell populations that may have stronger mesodermal than ectodermal predilections (Arsenian et al., 1998; Alipour et al., 2015; Lombana et al., 2015). Selection of cell immunophenotypes and subsequent culture in mesodermal and ectodermal basal medium will provide important information about the novel progenitor cells residing in the ectodermal-mesodermal interface. Nonetheless, the presence of mesodermal and ectodermal antigens and differentiation potential over multiple cell passages confirm that cells of both embryonic origins survived under the culture conditions employed in this study.

## Conclusions

Progenitor cell isolates from the ectodermal-mesodermal tissue interface of the equine hoof display characteristics of both tissue types and maintain progenitor qualities through multiple *in vitro* cell passages. The cells are present in both normal and inflamed tissue and key protein antigens, including those in cell to cell and cell to tissue junctions, are retained in culture. Hoof progenitor cells promote collagenous extracellular matrix formation on distinct scaffold carriers *in vivo*, confirming future opportunities for cell directed tissue formation. Results from this study set the stage for work to ascertain mechanisms by which inflammation impacts progenitor cells in the ectodermal-mesodermal niche. Methods to direct appropriate cell differentiation and tissue formation will provide new therapeutic approaches to inflammatory conditions of tissue interfaces.

## Ethics Statement

This study was approved by the Louisiana State University Animal Care and Use Committee prior to study initiation (protocol # 16-036).

## Author Contributions

QY and WD contributed to the study design, data collection and reduction, manuscript writing, and final approval of manuscript. VP and ML contributed to the study design, data collection and reduction, data analysis and interpretation, manuscript writing, and final approval of manuscript. EP, JD, and WR were responsible for data collection, manuscript writing, and final approval of manuscript.

### Conflict of Interest Statement

The authors declare that the research was conducted in the absence of any commercial or financial relationships that could be construed as a potential conflict of interest.
